# Evaluation of the implementation of the One Health approach in Guinea: Application of the NEOH framework to decentralized One Health platforms^☆^

**DOI:** 10.1016/j.onehlt.2026.101428

**Published:** 2026-04-24

**Authors:** Emile Faya Bongono, Castro Gbêmêmali Hounmenou, Stéphanie Maltais, Aminata Mbaye, Gnouma Laurent Koniono, Salifou Talassone Bangoura, Marc Yambayamba, Maladho Diaby, Alpha Kabinet Keita, Abdoulaye Touré, Alioune Camara, Youssouf Sidimé, Simon Rüegg

**Affiliations:** a Center for Research and Training in Infectious Diseases of Guinea, Hadja Mafory Campus, Gamal Abdel Nasser University of Conakry, Guinea; b Department of Veterinary Medicine, Chair of Infectious and Parasitic Diseases, Higher Institute of Science and Veterinary Medicine of Dalaba, Guinea; c Chair of Public Health and Pharmaceutical Policy, Department of Pharmaceutical and Biological Sciences, Faculty of Health Sciences and Technology, Gamal Abdel Nasser University of Conakry, Guinea; d Chair of Public Health, Department of Medical Sciences, Faculty of Health Sciences and Techniques, Gamal Abdel Nasser University of Conakry, Guinea; e Department of Computer Science, University of Labé, Guinea; f Department of Health Management, Evaluation and Policy, School of Public Health, University of Montreal, Canada; g Section of Epidemiology, Vetsuisse Faculty, University of Zurich, Zurich, Switzerland; h Centre de Recherche pour la Valorisation de l'Invention/Innovation Scientifique et Technologique en Guinée (CREVIST)

**Keywords:** One health, Zoonoses, Evaluation, NEOH, Guinea

## Abstract

Zoonotic diseases pose a major threat to human, animal, and environmental health, particularly in sub-Saharan Africa, where intersectoral coordination mechanisms remain limited. The One Health (OH) approach offers an integrated framework to strengthen surveillance, preparedness, and prevention of these threats. In Guinea, eight regionals OH platforms have been established; however, their performance has not been systematically evaluated. This study aimed to assess the systemic performance of these platforms and identify their strengths and weaknesses using the six dimensions of the Network for Evaluation of One Health (NEOH) framework.

A cross-sectional descriptive study was conducted across eight regions (*n* = 20 participants per region). The standardized NEOH questionnaire (0–1 scale) was completed individually. Dimension scores were aggregated using medians and interquartile ranges (IQR). The One Health Index (OHI) and the One Health Ratio (OHR) were calculated to assess overall integration and internal balance between operational and support components, respectively.

Marked regional heterogeneity was observed. N'zérékoré demonstrated the most balanced profile and the highest OHI value (0.33 [0.43]). In contrast, Boké, Kindia, and Mamou exhibited low scores in Sharing, Learning, and Systemic Organisation. Significant imbalances in OHR were observed in several regions, indicating misalignment between operational activities and structural support mechanisms.

Decentralized One Health platforms in Guinea display uneven levels of maturity. Strengthening information sharing, collective learning, and intersectoral governance mechanisms is essential to enhance systemic coherence and improve preparedness and response to priority zoonotic diseases**.**

## Highlights

• Assessment of One Health platforms in Guinea highlights strengths and challenges in zoonotic disease control.
• The Network for Evaluation of One Health (NEOH) framework enables structured analysis of One Health systems.
• Key gaps identified in data sharing and stakeholder coordination, indicating areas for improvement.
• Findings stress need for integrated approaches to strngthem One Health platforms and health system resilience.

## 1 Introduction

Emerging and re-emerging zoonotic diseases remain a persistent global threat at the interface of human, animal, and environmental health. More than 60% of known human infectious diseases are of animal origin, and approximately 75% of emerging pathogens originate from animals, underscoring the deep interconnection between species and ecosystems [1]. The increasing frequency and magnitude of zoonotic outbreaks such as Ebola virus disease in sub-Saharan Africa, avian influenza, coronavirus disease 2019 (COVID-19), and antimicrobial resistance illustrate the vulnerability of health systems when operating in a siloed manner [2], [3]. In low- and middle-income countries, particularly in sub-Saharan Africa, weak intersectoral collaborations, insufficient infrastructures, and fragile governance exacerbate vulnerability to health crises, leading to devastating social and economic consequences [4], [5], [6].

The One Health approach has emerged as an integrated response to address these complex challenges. It promotes multisectoral collaboration across human, animal, and environmental health sectors to improve disease prevention, detection, and response, while simultaneously strengthening sustainable health governance [7]. International institutions such as the World Health Organisation (WHO), Food and Agriculture Organisation (FAO) of the United Nations, World Organisation for Animal Health (WOAH), and United Nations Environment Programme (UNEP) have adopted One Health as a central pillar of global health security strategies [5], [8]. In recent years, many countries have begun to institutionalize this approach through national action plans, interministerial committees, and regional platforms aimed at coordinating surveillance and response to health threats [9]. However, despite its growing adoption, evaluating One Health implementation remains a methodological and practical challenge [10].

To address this gap, the Network for Evaluation of One Health (NEOH) has developed a conceptual and methodological framework to systematically assess One Health initiatives. The NEOH tool combines qualitative and quantitative indicators to analyze six dimensions: Reflection, Planning, Working, Sharing, Learning, and Systemic Organisation [9], [10]. By providing a structured assessment, the tool enables researchers to compare different initiatives, identify their strengths and weaknesses, and formulate recommendations to improve governance. The NEOH framework has been applied in Europe, Asia, and Africa to evaluate initiatives ranging from antimicrobial resistance control to zoonotic disease surveillance [11]. However, its application in sub-Saharan Africa remains limited, even though the institutionalization of One Health is relatively recent in the region. In Guinea, the need for an integrated approach is particularly critical. The country was the epicenter of the 2014–2016 Ebola virus disease outbreak, which revealed profound weaknesses in preparedness, coordination, and intersectoral communication [12], [13]. More recently, outbreaks of anthrax, rabies, Lassa fever, and avian influenza have highlighted the persistence of zoonotic risks in diverse contexts, both urban and rural [14], [15], [16]. In response to these threats, Guinea established a National One Health Platform (NOHP) and, within a decentralization framework, eight Regional One Health Platforms (ROHPs). These platforms bring together actors from the human, animal, and environmental health sectors, as well as representatives from civil society, administrative authorities, and technical and financial partners, to promote integrated governance at the local level [17].

Despite these advances, the implementation of One Health in Guinea still faces numerous obstacles. Limited resources, insufficient intersectoral communication, and disparities in stakeholder engagement undermine the performance and sustainability of the platforms [13], [15]. Moreover, few systematic evaluations have documented how these platforms function, their added value in surveillance and response, or the persistent gaps. In the absence of evidence, it is difficult to ensure accountability, mobilize resources, and inform decision-making at both national and international levels.

This study, therefore, addresses the lack of empirical data. Evaluating Guinea's regional One Health platforms using a standardized and internationally recognized tool such as NEOH not only highlights their strengths and weaknesses but also contributes to the comparative literature on One Health implementation. This approach is particularly timely as Guinea and other countries in the region strive to align their health governance systems with the Global Health Security Agenda (GHSA) and the International Health Regulations (IHR 2005), both of which emphasize multisectoral collaboration in preparedness and response [13], [18].

In a context characterized by limited resources and the gradual implementation of the One Health approach in Guinea, it is essential to rely on evaluation tools that focus not on the direct epidemiological impact of platforms, but on their organizational and functional maturity. From this perspective, the NEOH evaluation framework was applied to the eight existing One Health platforms in the country to examine how they operationalize One Health principles, their capacity to support integrated surveillance and coordinated response, and the systemic conditions required for effective intersectoral collaboration.

This work represents one of the first applications of the NEOH tool in Guinea and pursues two main objectives: (i) to analyze the implementation of the One Health approach through One Health platforms operating at the decentralized level; and (ii) to identify the strengths and weaknesses of these platforms in terms of multisectoral collaboration and systemic organisation, with a view to strengthening One Health governance. By enabling an assessment of the actual functioning of the platforms and highlighting key leverage points for improvement, this approach aims to inform public policy decisions and contribute to international discussions on the effective operationalization of One Health in resource-limited settings.

## 2 Materials and methods

### 2.1 Study setting

**Fig. 1:**
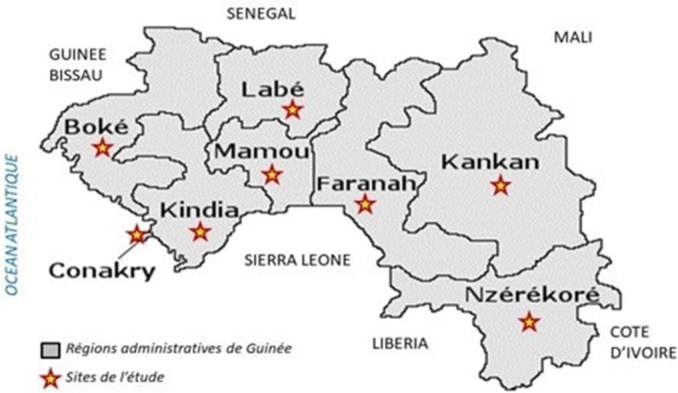
Administrative map of the Republic of Guinea showing the study sites.

In Guinea, the study was conducted in Conakry and in seven sentinel sites corresponding to the prefectures of Boké, Kindia, Labé, Mamou, Faranah, Kankan, and Nzérékoré **(**Fig. 1**).** These sites were selected because of their epidemiological relevance, characterized by the presence of zoonotic and endemic diseases and by close interactions between human populations, animals, and their environment. Their geographic distribution covers all regions of the country, thereby providing a range of ecological and socio-institutional contexts relevant for the analysis of One Health platforms.

### 2.2 Study type

A descriptive cross-sectional study combined with a participatory research approach was conducted among stakeholders of the eight ROHPs in Guinea.

### 2.3 Participant selection

The study population consisted of actors from ROHPs in Guinea, including veterinarians, healthcare professionals, environmental specialists, and support actors. Support actors included territorial administrators, local elected officials, media representatives, hunters, religious leaders, community health workers in human, animal and environmental health, traditional healers, farmers, and technical and financial partners. The participatory workshops were conducted in French, with clarifications provided in local languages when needed. In each of the eight regions, One Health platform focal points helped identify 20 participants (160 in total), ensuring representation across actor categories: 10 from the human health sector, 3 from the animal health sector, 4 from livestock production, and 3 from supporting sectors. The number of participants per region was set at twenty due to logistical and financial constraints. The organisation of the workshops required covering participants' transportation and catering costs, which limited the feasibility of larger group sizes. In addition, the number of actors directly involved in regional One Health platforms is relatively limited and generally does not exceed twenty individuals. Finally, the participatory workshop format required conditions conducive to effective interaction and facilitation, which become increasingly difficult to maintain as group size increases, particularly because of constraints related to available space and group dynamics.

### 2.4 Data collection

The evaluation was conducted using the standard NEOH evaluation framework (NEOH Handbook, 2018 version). The original Excel-based scoring tool was used without modification of the core structure or scoring scale (0–1). Minor contextual adaptations were made to clarify terminology and ensure relevance to the Guinean context, while preserving the conceptual integrity of the framework**.**

Researchers administered the NEOH questionnaire to all participants during three-day participatory workshops held in each of the eight regions. Each stakeholder assigned a score for each dimension, based on their perception and level of involvement in the ROHPs. The NEOH tool, presented in the form of an Excel matrix, was used to record these individual scores. The questionnaires were pre-tested to ensure their relevance and reliability. For each dimension, a score ranging from 0 to 1 was assigned: 0 indicated a complete absence of One Health integration (sectoral silo approaches), while 1 reflected full integration of One Health principles, representing an ideal state of strong interdisciplinary collaboration, systemic thinking, and coordinated action across sectors.

Each regional workshop was facilitated by trained moderators familiar with the NEOH framework. After presentation of each question, participants discussed the item collectively before assigning scores individually. When substantial discrepancies emerged, facilitated discussions were conducted to reach a shared understanding of the scoring criteria. The NEOH questionnaire was completed individually by each participant. Collective discussions were conducted solely to ensure a shared understanding of the questions and scoring criteria, without imposing consensus on the scores assigned. This approach preserved the independence of responses while ensuring methodological consistency.

### 2.5 Data processing and analysis

In the first step, raw data from the questionnaires were entered and organized in Microsoft Excel (Office 2016). Subsequently, using scripts developed in R software (version 3.5.3), we: (i) extracted the individual scores from each participant, (ii) calculated mean scores by region for each of the six dimensions, and (iii) computed the One Health Index (OHI) and One Health Ratio (OHR) for each region based on these mean scores. Dimension-specific scores (Thinking = ScT, Planning = ScP, Working = ScW, Sharing = ScS, Learning = ScL, and Systemic Organisation = ScO) were calculated based on individual participants' responses to the standardized questions of the NEOH evaluation framework. Each question was scored on an ordinal scale ranging from 0 to 1, in accordance with NEOH guidelines. In a first step, for each participant, the scores assigned to the questions were summarized by calculating the median of the questions associated with each dimension, thereby yielding an individual score per dimension. This step ensures appropriate treatment of the ordinal nature of the data and reduces the influence of extreme values.

In a second step, individual dimension scores were aggregated at the regional level by calculating, for each dimension, the median of the individual scores across all participants in the corresponding region. This hierarchical approach (question → individual → gouvernorat) produces robust and representative regional scores reflecting the functioning of One Health platforms. The resulting regional scores, ranging from 0 to 1, served as the basis for the calculation of the composite One Health indicators, including the OHI and OHR. In a second step, individual dimension scores were aggregated at the regional level by calculating, for each dimension, the median of the individual scores across all participants in the corresponding region. This hierarchical approach (question → individual → region) produces robust and representative regional scores reflecting the functioning of One Health platforms. The resulting regional scores, ranging from 0 to 1, served as the basis for the calculation of the composite One Health indicators, including the OHI and the OHR [19]. These visualizations make it possible to represent both the operational aspects of One Health (reflection, planning, working) and the supporting infrastructures (sharing, learning, systemic organisation). The surface area of the radar chart reflects the level of each dimension and is used to calculate the OHI, ranging from 0 to 1, and the OHR, which assesses the balance between One Health operations and infrastructures. The calculation of these indices followed the formulas proposed by Rüegg et al. [20], [21].

(1) $$\mathrm{OHI}=\frac{\left\{\left(\mathrm{ScP}\times \mathrm{ScT}\right)+\left(\mathrm{ScL}\times \mathrm{ScP}\right)+\left(\mathrm{ScS}\times \mathrm{ScL}\right)+\left(\mathrm{ScO}\times \mathrm{ScS}\right)+\left(\mathrm{ScW}\times \mathrm{ScO}\right)+\left(\mathrm{ScT}\times \mathrm{ScW}\right)\right\}}{6}$$

Where ScP is the score obtained for Planning; ScT is the score obtained for Reflection; ScW is the score obtained for Working; ScO is the score obtained for Systemic Organisation; ScL is the score obtained for Learning; and ScS is the score obtained for Sharing. Thus, if the value of this index is less than 1, it indicates an imbalance between operational and support aspects, whereas a value equal to 1 indicates a balance between the two.

The OHR reflects the relative contribution of operational aspects and support aspects, indicating the balance between operational inputs and supporting infrastructures [19].

(2) $$\mathrm{OHR}=\frac{\left(\frac{\mathrm{ScO}\times {\mathrm{ScW}}^{2}}{\mathrm{ScO}+\mathrm{ScW}}\right)+\left(\mathrm{ScW}\times \mathrm{ScT}\right)+\left(\mathrm{ScT}\times \mathrm{ScP}\right)+\left(\frac{{\mathrm{ScP}}^{2}\times \mathrm{ScL}}{\mathrm{ScP}+\mathrm{ScL}}\;\right)}{\left(\frac{\mathrm{ScP}\times {\mathrm{ScL}}^{2}}{\mathrm{ScP}+\mathrm{ScL}}\right)+\left(\mathrm{ScL}\times \mathrm{ScS}\right)+\left(\mathrm{ScS}\times \mathrm{ScO}\right)+\left(\frac{{\mathrm{ScO}}^{2}\times \mathrm{ScW}}{\mathrm{ScO}+\mathrm{ScW}}\;\right)}$$

In addition to regional medians, interquartile ranges (IQR) were calculated to assess within-region variability in scoring, providing insight into the degree of consensus among participants.

Given the ordinal nature of the NEOH scoring system (scale 0–1), descriptive statistics were based on the median and interquartile range (IQR). For each dimension and region, the median of individual scores was calculated to represent central tendency, while the IQR (difference between the 75th and 25th percentiles) was used to quantify within-region variability.

The use of the IQR provides insight into the degree of consensus among participants within the same region. A low IQR indicates homogeneity of perceptions and relative agreement regarding platform performance, whereas a high IQR reflects heterogeneity in responses and may suggest uneven implementation of One Health mechanisms within the region.

The One Health Index (OHI) was calculated using the six NEOH dimensions according to the recommended formula. The OHI is bounded between 0 and 1 and reflects the overall level of integration between operational and structural components. Values approaching 1 indicate coherent and balanced integration across dimensions, whereas lower values suggest partial or insufficient integration.

The One Health Ratio (OHR) was calculated to assess the internal balance between operational dimensions (Thinking, Planning, and Working) and support dimensions (Sharing, Learning, and Systemic Organisation). Unlike the OHI, the OHR represents a ratio and is therefore not bounded by 1. A value of 1 was used as a theoretical reference point indicating equilibrium between operational and structural components. Values above 1 indicate predominance of operational activities relative to supporting mechanisms, while values below 1 suggest stronger structural components compared to operational implementation. Because the ratio may be sensitive to low denominator values, OHR results were interpreted jointly with individual dimension scores to avoid overinterpretation.

### 2.6 Ethical considerations

The study was approved by the National Health Research Ethics Committee (CNERS) under authorization number 025/CNERS/23.

## 3 Results

Table 1 presents the regional median scores and interquartile ranges (IQR) for the six NEOH dimensions, together with the One Health Index (OHI) and the One Health Ratio (OHR). Marked heterogeneity was observed across regions, both in terms of central tendency and within-region variability.

**Table 1:** Median and interquartile range (IQR) of NEOH dimension scores, One Health Index (OHI), and One Health Ratio (OHR) across decentralized One Health platforms in Guinea.

Region	ScT [IQR]	ScP [IQR]	ScS [IQR]	ScW [IQR]	ScL [IQR]	ScO [IQR]	OHI [IQR]	OHR [IQR]
Boké	0.00 [0.25]	0.00 [0.30]	0.00 [0.28]	0.00 [0.17]	0.00 [0.10]	0.00 [0.40]	0.00 [0.02]	0.99 [1.64]
Conakry	0.00 [0.65]	0.00 [0.77]	0.00 [0.14]	0.00 [0.22]	0.04 [0.09]	0.15 [1.00]	0.03 [0.14]	0.80 [0.88]
Faranah	0.70 [1.00]	0.20 [1.00]	0.00 [0.50]	0.05 [1.00]	0.21 [0.52]	0.00 [1.00]	0.08 [0.35]	1.55 [3.70]
Kankan	0.00 [0.20]	0.42 [0.62]	0.00 [0.25]	0.40 [1.00]	0.31 [0.34]	1.00 [0.85]	0.14 [0.24]	1.10 [1.14]
Kindia	0.00 [0.00]	0.00 [0.00]	0.00 [0.00]	0.00 [0.00]	0.43 [0.45]	0.00 [0.00]	0.00 [0.00]	3.49 [1.16]
Labe	0.25 [1.00]	0.00 [0.92]	0.00 [0.82]	0.00 [1.00]	0.01 [0.28]	0.00 [1.00]	0.15 [0.33]	1.66 [2.11]
Mamou	0.00 [0.40]	0.00 [0.10]	0.00 [0.00]	0.00 [0.45]	0.06 [0.27]	0.00 [1.00]	0.00 [0.22]	2.14 [2.00]
N'zérékoré	0.80 [0.75]	0.50 [1.00]	0.55 [1.00]	1.00 [0.92]	0.46 [1.00]	1.00 [0.62]	0.33 [0.43]	1.00 [0.80]

Note: Values are presented as median [interquartile range (IQR)]. Scores range from 0 to 1, with higher values indicating stronger implementation of the respective One Health dimension. The One Health Index (OHI) reflects the overall level of integration across the six dimensions, while the One Health Ratio (OHR) represents the internal balance between operational (Thinking, Planning, Working) and support (Sharing, Learning, Systemic Organisation) components. An OHR value close to 1 indicates relative equilibrium between these components.

Disparities were evident in the operational dimensions (Thinking, Planning, and Working). N'zérékoré exhibited the highest median scores across most operational components (ScT = 0.80; ScP = 0.50; ScW = 1.00), suggesting a comparatively advanced level of intersectoral engagement and structured implementation of the One Health approach. In contrast, Boké, Conakry, Kindia, and Mamou displayed median values close to zero for most operational dimensions. However, the relatively wide IQRs observed in some regions (e.g., Conakry: ScP [0.77], ScT [0.65]) indicate substantial variability in participant perceptions, reflecting heterogeneous implementation within the same platform.

Support dimensions (Sharing, Learning, and Systemic Organisation) appeared generally weaker and less consistent. N'zérékoré stood out with relatively high medians in Sharing (0.55) and Systemic Organisation (1.00), suggesting better-structured coordination mechanisms.

The One Health Index was highest in N'zérékoré (0.33 [0.43]), indicating comparatively stronger coherence among the evaluated dimensions. Similarly, the One Health Ratio in N'zérékoré was close to 1 (1.00 [0.80]), reflecting a relative balance between operational activities and organizational infrastructure

**Analysis of radar profiles of the regional One Health platforms**

**Fig. 2:**
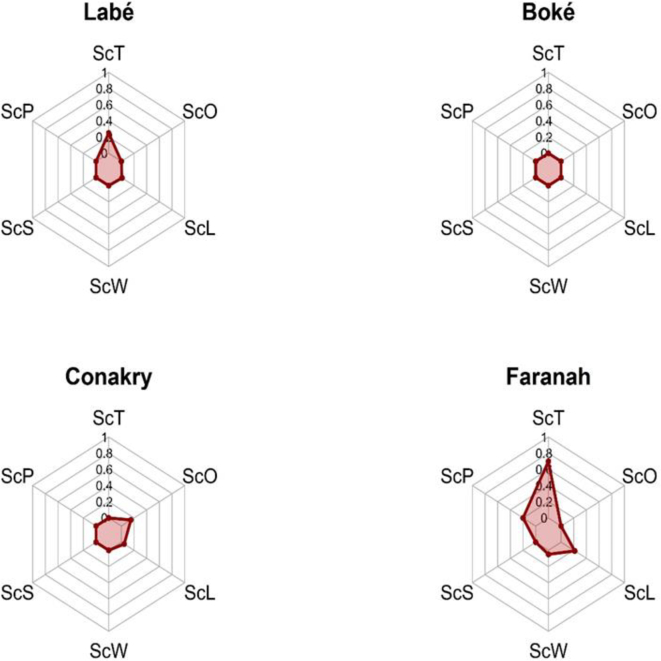
Comparative radar representation of median NEOH dimension scores across decentralized One Health platforms in Guinea (Labé, Boké, Conakry, Faranah), illustrating regional variability in organizational maturity and systemic coherence. Values represent regional medians (scale 0–1). The six dimensions include Thinking (ScT), Planning (ScP), Working (ScW), Sharing (ScS), Learning (ScL), and Systemic Organisation (ScO). Higher scores indicate stronger integration of the respective One Health dimension.

**Fig. 3:**
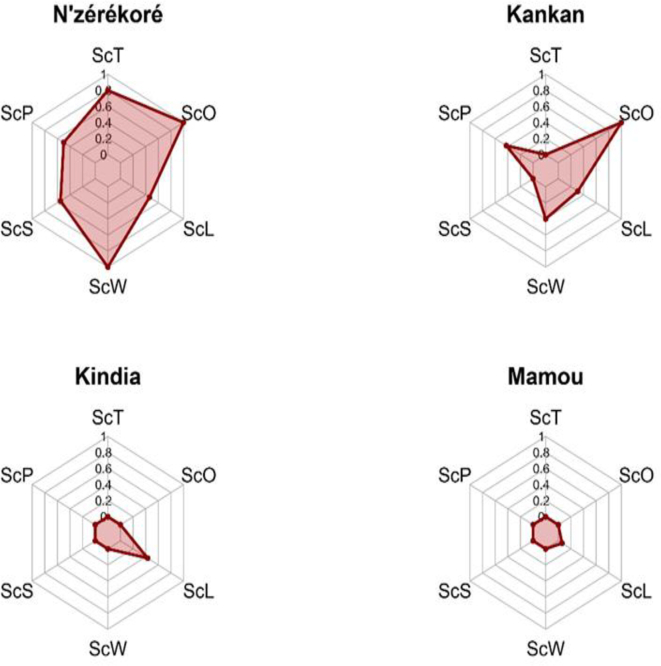
Radar representation of median scores for the six NEOH dimensions across four decentralized One Health platforms in Guinea (N'zérékoré, Kankan, Kindia, Mamou).

The radar plots highlighted regional differences in the configuration of the six NEOH dimensions. N'zérékoré displays the most balanced profile and the highest scores across both operational dimensions (Thinking, Planning, and Working) and support dimensions (Sharing, Learning, and Systemic Organisation) (Fig. 2). In contrast, Boké and Conakry show low median scores across most dimensions (Fig. 3). Overall, these findings underscore unequal levels of maturity among decentralized One Health platforms and identify clear priorities for strengthening governance and intersectoral collaboration mechanisms.

## 4 Discussion

The findings revealed marked heterogeneity across regions in terms of organizational maturity and systemic coherence. This variability is consistent with observations reported in other sub-Saharan African countries, where the implementation of the One Health approach remains strongly influenced by institutional context and the availability of resources [22], [23], [24].

The low scores observed in the Sharing dimension across nearly all eight regions highlight the insufficient formalization of data and information exchange mechanisms between sectors. Such fragmentation of surveillance systems represents a well-recognized barrier to the effective operationalization of the One Health approach [7], [25]. Strengthening regional platforms should include the institutionalization of multisectoral data review meetings, the development of formal information-sharing protocols, and the implementation of integrated digital tools. Experiences from Tanzania and Uganda have demonstrated that structured coordination frameworks significantly enhance the early detection of zoonotic threats [26], [27].

Low scores in the Learning dimension, particularly in Boké, Conakry, Labé, and Mamou, highlight the absence of systematic feedback mechanisms and joint training processes. The importance of organizational learning for strengthening health system resilience has been demonstrated in several post-epidemic contexts [28], [29]. In Guinea, the 2014–2016 Ebola virus disease outbreak underscored the necessity of sustained multisectoral coordination beyond emergency response situations [30], [31]. Institutionalizing joint simulation exercises and systematic after-action reviews would help transform ad hoc coordination into a structured and sustainable preparedness mechanism.

The weaknesses observed in the Systemic Organisation dimension across regions reflect, in part, unclear mandates and insufficient integration of the environmental sector. Similar challenges have been documented in Mali and Burkina Faso, where the absence of formalized engagement of environmental authorities has limited the internal coherence and effectiveness of One Health platforms [32], [33]. The adoption of regional decrees clearly defining roles and responsibilities, together with the allocation of dedicated budget lines for coordination, could strengthen institutional stability and enhance the sustainability of One Health platforms.

The Guinean epidemiological context further reinforces the relevance of these findings. The forest region of N'zérékoré has been the epicenter of Ebola outbreaks, underscoring the critical need for well-coordinated, multisectoral preparedness and response mechanisms at the decentralized level [12]. The management of such crises requires effective coordination between wildlife surveillance, veterinary services, and community health systems, which directly corresponds to the dimensions assessed by the NEOH framework. Rabies remains endemic in sub-Saharan Africa, with transmission occurring predominantly through domestic dogs, further highlighting the importance of integrated planning, intersectoral coordination, and sustained collaboration between public health and veterinary authorities [34]. Effective rabies control requires intersectoral planning, mass animal vaccination campaigns, and community awareness initiatives elements that are directly dependent on strong Sharing and Planning mechanisms. Similarly, episodes of highly pathogenic avian influenza reported in the regions of Kindia and Labé have underscored the importance of integrated animal–human surveillance systems to ensure timely detection and coordinated response [35], [36]. Regions such as Faranah, Kindia, Labé, and Mamou, which exhibited marked imbalances in the OHR, may face challenges in effectively coordinating these multisectoral actions. Such imbalances suggest a misalignment between operational activities and the structural support required to sustain them.

These findings have direct implications for national health security. Guinea is committed to the implementation of the International Health Regulations (IHR 2005), which emphasize multisectoral coordination and integrated surveillance capacities as core components of preparedness and response [37] Guinea has also participated in Joint External Evaluations (JEE) [38]. Strengthening decentralized One Health platforms would contribute to improving the core capacities required under the IHR framework and would support the objectives of the Global Health Security Agenda by enhancing multisectoral preparedness, coordination, and response at the subnational level [39], [40].

The NEOH framework provides an operational tool to identify priority regions for strategic investment, including Kindia, Labé, Mamou, Boké, Conakry, and Faranah. The combined interpretation of the One Health Index (OHI), reflecting the level of integration, and the One Health Ratio (OHR), reflecting internal balance, offers a relevant indicator to guide decision-makers toward targeted and context-specific interventions.

The regional sample size (*n* = 20 per region) may limit generalizability and introduce potential selection and social desirability biases. However, this size reflects logistical and financial constraints related to workshop organisation, including transport reimbursement, venue rental, and accommodation for non-resident participants. Moreover, the number of formally engaged One Health actors at the decentralized level remains relatively limited in most regions. The selected sample therefore represents a substantial proportion of available stakeholders. Questionnaires were completed individually to minimize response bias.

## 5 Conclusion

This study highlighted unequal levels of maturity among decentralized One Health platforms in Guinea. While certain regions, particularly N'zérékoré, demonstrate relatively coherent integration of operational and structural dimensions, others reveal significant gaps in information sharing, collective learning, and systemic organisation. The combined use of the One Health Index (OHI) and the One Health Ratio (OHR) enables the identification of internal imbalances and priority areas for strengthening at the subnational level.

Institutional strengthening of coordination mechanisms, formal integration of the environmental sector, and the establishment of sustainable information-sharing systems are essential to improve platform coherence and effectiveness. By consolidating these dimensions, Guinea could enhance its capacity to prevent and respond effectively to priority zoonotic diseases, while strengthening overall national health security.

## CRediT authorship contribution statement

**Emile Faya Bongono:** Writing – review & editing, Writing – original draft, Methodology, Investigation, Formal analysis, Data curation, Conceptualization. **Castro Gbêmêmali Hounmenou:** Methodology, Investigation, Formal analysis, Conceptualization. **Stéphanie Maltais:** Writing – review & editing, Visualization, Validation, Resources. **Aminata Mbaye:** Methodology, Investigation, Conceptualization. **Gnouma Laurent Koniono:** Visualization, Methodology. **Salifou Talassone Bangoura:** Methodology. **Marc Yambayamba:** Formal analysis. **Maladho Diaby:** Visualization. **Alpha Kabinet Keita:** Resources, Funding acquisition. **Abdoulaye Touré:** Supervision, Resources, Funding acquisition. **Alioune Camara:** Supervision, Project administration, Funding acquisition. **Youssouf Sidimé:** Supervision, Validation. **Simon Rüegg:** Validation, Supervision, Methodology, Conceptualization.

## Funding statement

This research was conducted with financial support from the 10.13039/501100000193International Development Research Centre (IDRC) as part of the thesis work of Emile Faya Bongono. It was carried out within the framework of the project Decentralize and Make Operational the “One Health” Platforms (DOPERAUS), under grant number 109812.

## Declaration of competing interest

The authors declare that they have no financial interests or personal relationships that could have influenced the work reported in this article.

This research was funded by the International Development Research Centre (IDRC, Canada) through the DOPERAUS project (Grant No. 109812), as part of the Collaborative One Health Research Initiative on Epidemics (COHRIE). The funder had no role in the design of the study, data collection, analysis, or interpretation of the results.

## Data Availability

Data will be made available on request.
